# Effect of dolutegravir on ferritin, iron, and C-reactive protein among people living with HIV and co-infections

**DOI:** 10.4102/sajhivmed.v25i1.1543

**Published:** 2024-04-30

**Authors:** Bridget Kamurai, Raylton P. Chikwati, Donald Vhanda, Terrence Nyamayaro, Justen Manasa, Vinie Kouamou

**Affiliations:** 1 Department of Chemical Pathology, Faculty of Medicine and Health Sciences, University of Zimbabwe, Harare, Zimbabwe; 2 Department of Chemical Pathology, Faculty of Health Sciences, University of the Witwatersrand, Johannesburg, South Africa; 3 Sydney Brenner Institute for Molecular Bioscience, Faculty of Health Sciences, University of the Witwatersrand, Johannesburg, South Africa; 4 Department of Internal Medicine, Faculty of Medicine and Health Sciences, University of Zimbabwe, Harare, Zimbabwe

**Keywords:** HIV, co-infections, ferritin, iron, virological outcomes, dolutegravir

## Abstract

**Background:**

Dolutegravir-based antiretroviral therapy (ART) is currently recommended as the preferred first-line ART in many resource-limited settings. However, little is known about the clinical experience of dolutegravir within a context of prevalent co-infections.

**Objectives:**

To assess virological outcomes, and iron, ferritin and C-reactive protein (CRP) levels among people living with HIV (PLWH) and co-infections after initiating or re-initiating dolutegravir-based ART.

**Method:**

This prospective study was conducted between August 2022 and August 2023. Study participants were recruited from an HIV opportunistic infection clinic. Screening for co-infections (syphilis, hepatitis B virus, cytomegalovirus and herpes simplex virus) was performed at baseline, prior to ART initiation. Plasma HIV viral load (VL), CRP, ferritin and iron levels were measured at baseline and at the 6-month follow-up period.

**Results:**

A total of 100 participants (51 women and 49 men) were enrolled in this study. The median age of the participants was 39 years. The prevalence of co-infections was 30%. Prior to ART initiation, participants with co-infections had higher VL, CRP and ferritin, and lower iron levels, compared to those without co-infections (*P* < 0.001). Following 6 months of ART, CRP and ferritin levels decreased while iron levels increased, regardless of co-infection status. However, CRP and ferritin remained significantly higher in those with co-infections despite similar and high rates of virologic suppression in both groups.

**Conclusion:**

The presence of co-infections in PLWH is associated with higher VL and with chronic inflammation. Ferritin and CRP decreased on dolutegravir-based ART but remained higher in people with co-infections despite similar rates of virologic suppression.

**What this study adds:** Co-infections among people living with HIV were associated with higher viral loads, CRP and ferritin. Inflammatory markers decreased on dolutegravir-based ART but remained marginally higher in people with co-infections despite similar rates of virologic suppression.

## Introduction

The high prevalence of HIV continues to be a major global health problem, particularly in sub-Saharan Africa (SSA).^1^ According to the Joint United Nations Programme report on HIV/AIDS, in 2022, an estimated 39 million people were living with HIV (PLWH) and approximately 630 000 people died from AIDS-related illnesses globally.^1^ The high prevalence of sexually transmitted infections (STIs) in many resource-limited settings (RLS) presents more problems with prevention and treatment strategies for HIV/AIDS.^2,3^ The association between HIV and STI has been widely characterised. In South Africa, Kharsany et al. showed a bidirectional relationship between STIs (HSV-2, chlamydia and gonorrhoea) and HIV viral load (VL) among PLWH,^2^ thus contributing to sustained STI and HIV burden.^2^ Another study from rural Uganda showed that the prevalence of STIs (chlamydia, gonorrhoea and syphilis) was still high, despite a 90% VL suppression.^3^ Co-infections with hepatitis B virus (HBV), syphilis, cytomegalovirus (CMV) and herpes simplex virus (HSV) have been associated with worse clinical outcomes in PLWH.^4,5,6,7,8^

Studies have shown that HIV and some co-infections (CMV and HBV) are associated with increased systemic inflammation.^9,10^ The systemic inflammation activates HIV replication in latent cells and recruitment of uninfected cells, thereby sustaining inflammation and further HIV replication.^9,10^ Furthermore, studies have reported an association between chronic inflammation and the development of cardiometabolic diseases such as hypertension, stroke, and diabetes,^11^ which are prevalent non-communicable diseases in SSA.^12^ In addition, HIV independently increases inflammation through the interaction of its viral proteins, trans-activator of transcription, virion-associated protein, and glycoprotein 120 on host cells.^13,14,15^

In the context of HIV infection, anaemia is caused by several factors including co-existing infections and iron deficiency.^16,17,18^ The prevalence of anaemia among PLWH has been reported to be as high as 80% to 90% in some settings.^19,20^ Anaemia is often associated with the severity of HIV infection, predicting HIV/AIDS progression and mortality among PLWH.^19,21^ However, the use of antiretroviral therapy (ART) has been shown to reduce the prevalence of anaemia.^19^ Previous studies showed that iron deficiency and anaemia were prevalent among Kenyan and Ghanaian adults living with HIV.^22,23^ Data on the levels of iron, ferritin and C-reactive protein (CRP) among PLWH in RLS are limited.

Despite the vast literature on the impact of co-infections in the aetiology of HIV, there is a lack of data in SSA, where the prevalence of HIV remains high. Furthermore, there is limited understanding regarding the clinical outcomes of PLWH and co-infections, who begin ART based on dolutegravir in RLS. This study therefore aimed to assess virological outcomes, iron, ferritin and CRP levels among PLWH and co-infections, after initiating a dolutegravir-based ART.

## Research methods and design

### Study design, setting and population

In this prospective cohort study, we recruited ART-naïve PLWH and individuals reinitiating first-line ART after reporting previous exposure to ART but having defaulted on ART for at least 3 months. Participants were adults (≥ 18 years) who were attending care at an HIV opportunistic infection clinic. All participants were recruited and followed up between August 2022 and August 2023.

### Study procedure

Individuals seeking care at the clinic were consecutively invited to participate in the study. Sociodemographic characteristics (age, gender, educational status, marital status) and ART history data were obtained through an interview-based questionnaire and also from the clinic’s electronic medical records. At baseline, trained nurses collected blood specimens to screen for HBV, syphilis, CMV and HSV, and to measure HIV VL, CRP, ferritin and iron levels. All participants were seen after a median duration of 27 (25–30) weeks, where blood specimens were again collected for HIV VL, CRP, ferritin and iron follow-up measurements.

### Laboratory measurements

Screening for syphilis, HSV, hepatitis B surface antigen (HBsAg) and CMV antibodies was done using rapid test kits, according to manufacturer’s instructions (Chek Acrobiotech, Rancho Cucamonga, California, United States [US]). All of the rapid tests used in this study followed the lateral flow chromatographic immunoassay test principle. For syphilis screening, both treponemal and non-treponemal (Rapid Plasma Reagin [RPR]) detection were screened. A positive result on both tests was considered positive for syphilis. For inconclusive results, a tie breaker (Venereal Disease Research Laboratory test [VDRL]) was done. All participants were screened for HSV-1 and -2 IgG and IgM antibodies. Positivity on one of the antibodies was considered HSV positive. The same principle was applied for CMV antibody detection.

Plasma ferritin was assayed on the Maglumi Snibe X3 analyser (Shenzhen New Industries Biomedical Engineering Co., Ltd, Guangdong, China) based on the sandwich immunoluminometric assay test principle. The measuring range of the assay was 0 ng/mL to 2000 ng/mL and the coefficient of variation for ferritin was 9.44%.

The Biobase chemistry analyser (Biobase Biotech Co, Jinan, China) was used for the colorimetric quantification of plasma iron. The measuring range for the assay was 0 µmol/L to 180 µmol/L and the coefficient of variation was < 10%. CRP was measured on the Getein analyser according to the manufacturer’s instructions (Getein Biotech, Nanjing, China). The measuring range for CRP was 0.5 mg/L to 200 mg/L and the coefficient of variation was < 10%. However, manual dilution was done for samples with a CRP level of ≥ 200 mg/L and the value obtained after dilution was multiplied by the dilution factor.

### Statistical analyses

All statistical analyses were performed using Stata version 17.0 (StataCorp LLC, College Station, Texas, US). Non-parametric continuous data were expressed as medians and interquartile ranges (IQR). The unpaired Mann-Whitney U test was used to explore differences between the study variables VL, CRP, iron, and ferritin levels in PLWH, with and without co-infections, at baseline. In addition, the Wilcoxon paired test was used to explore differences in the study variables at the follow-up visit. The Fisher’s exact and Chi-square tests were used in assessing differences between categorical variables in PLWH, with and without co-infections, as appropriate. The level of significance was set at a threshold of *P*-value < 0.05.

The sample size was calculated using the likelihood-ratio test comparing two independent proportions (proportions of virological outcomes among PLWH, with co-infection [~26%]^24^ and without co-infection [~6.3%]), for cohort studies on Stata/SE 17.0 (StataCorp, College Station, Texas, US). The estimated sample size was 100.

### Ethical considerations

Ethical approval was obtained from the Joint Research and Ethics Committee of the University of Zimbabwe (JREC/303/2022). All study participants were asked to provide written informed consent. Participant data were deidentified and password protected to protect confidentiality.

## Results

### Baseline characteristics of the participants

We enrolled 100 participants. The median (IQR) age of the participants was 39 years (31 years to 48 years). Half of the participants, 51% (*n* = 51/100) were women (Table 1). Most of the participants were married (52%), had attended secondary education (88%), and were unemployed (57%) (Table 1). Eighty-seven per cent of the study participants were ART naïve and 13% had previously defaulted treatment. Half of the participants (50%, *n* = 48/96) with available baseline CD4 cell count data had advanced HIV disease, with CD4 < 200 cells/mm^3^. Baseline CD4 cell count was significantly lower in participants with co-infection compared to those without co-infection. There were no differences between participants with co-infections and those without in relation to age, sex, marital, educational and employment status (Table 1).

**TABLE 1:** Baseline sociodemographic characteristics of the participants.

Characteristics	Total (*N* = 100)				HIV mono-infection (*n* = 70)				HIV/co-infections (*n* = 30)				*P*
	Median	IQR	*n*	%	Median	IQR	*n*	%	Median	IQR	*n*	%	
**Age (years)**	39	31–48	-	-	38	31–46	-	-	43	29–52	-	-	0.436
CD4 cell count, cells/mm^3^	190	79–357	-	-	263	126–452	-	-	102	36–167	-	-	< 0.001
**Gender**													
Male	-	-	49	49.0	-	-	32	45.7	-	-	17	56.7	0.315
Female	-	-	51	51.0	-	-	38	54.3	-	-	13	43.3	-
**Marital status**													
Married	-	-	52	52.0	-	-	40	57.1	-	-	12	40.0	0.157
Non-married	-	-	48	48.0	-	-	30	42.9	-	-	18	60.0	-
**Educational status**													
Primary	-	-	4	4.0	-	-	3	4.3	-	-	1	3.3	0.129
Secondary	-	-	96	96.0	-	-	67	95.7	-	-	29	96.7	-
**Employment status**													
Employed	-	-	43	43.0	-	-	29	41.4	-	-	14	46.7	0.223
Unemployed	-	-	57	57.0	-	-	41	58.6	-	-	16	53.3	-

Note: Co-infection was defined as having HIV plus syphilis and/or hepatitis B virus and/or cytomegalovirus and/or herpes simplex virus. Non-married individuals were either single, divorced or widowed.

IQR, interquartile range.

### Prevalence of co-infections

From the total study population, 30% (*n* = 30/100) had at least one co-infection: syphilis (14%), HBV (10%), HSV (6%), and CMV (3%). From these 30 participants, two (7%) had both syphilis and HBV and were subsequently given benzathine penicillin and tenofovir disoproxil fumarate (TDF) treatment during the study.

### Viral load, C-reactive protein, iron, and ferritin at baseline and follow-up

At baseline, the median VL was significantly higher in participants who had co-infections than those without (Table 2). Similarly, plasma levels of CRP and ferritin were significantly higher in participants with co-infections than those without. Plasma iron levels were significantly lower in participants with co-infections than those without (Table 2).

**TABLE 2:** Clinical outcomes at baseline and 6-month follow-up.

Characteristics	Co-infection	Pre-ART (baseline)		Post-ART (follow-up)	
		Median	IQR	Median	IQR
VL (copies/mL)	Absent	73 200	11 200–195 000*	26.0	0.0–30.0**
	Present	710 000	530 000–1 312 000	30.0	20.0–30.0**
CRP (ng/mL)	Absent	90.5	46.3–156.0*	3.6	2.4–6.3**
	Present	872.5	606.0–1302.0	5.2	3.5–12.6**
Iron (μmol/L)	Absent	11.2	7.6–14.6*	14.2	12.2–17.5**
	Present	3.2	2.0–3.8	12.4	9.2–13.8**
Ferritin (ng/mL)	Absent	156.0	96.0–196.0*	94.5	72.0–141.0**
	Present	2260.0	624.0–4890.0	136.0	85.0–212.0**

Note: Not detected where viral load is 0. Comparisons at baseline performed by the unpaired Mann-Whitney test and paired comparisons between baseline and follow-up measurements performed by the paired Wilcoxon test. Normal reference ranges for CRP: 0 ng/mL – 3 ng/mL, Iron: 7.6 µmol/L – 31.3 µmol/L, ferritin: 6.90 ng/mL – 282.5 ng/mL.

ART, antiretroviral therapy; CRP, C-reactive protein; IQR, interquartile range; VL, viral load.

* , *P* < 0.001: Absence versus presence of co-infection at baseline.

** , *P* < 0.001: Baseline versus follow-up measurements.

Six months after ART initiation or reinitiation, we observed significant decreases in levels of HIV VL, CRP and ferritin, and increased levels of plasma iron among participants with co-infections (Table 2). The direction and magnitude of these changes were also observed in participants without co-infections.

Six months after initiating ART, the presence of co-infections was associated with significantly higher levels of CRP (5.2 [3.5–12.6] vs 3.6 [2.6–7], *P* = 0.019) and ferritin (136 [85–212] vs 94.5 [72–141], *P* = 0.019) than the absence of co-infections. Levels of plasma iron remained significantly lower among participants with co-infection at follow-up (12.4 [9.2–13.8] vs 14.2 [12.2–17.5], *P* < 0.001) than those without co-infections. Six months after ART initiation, virological suppression (VL < 1000 copies/mL) was high (99%) among all participants, with no significant differences in the levels of HIV VL according to co-infection status.

## Discussion

In this study, we assessed changes in HIV VL, iron, ferritin, and CRP among PLWH and co-infections following 6-month initiation on a dolutegravir-based ART. Co-infections were associated with higher VL, CRP, and ferritin, but decreased iron levels at baseline. After 6 months of ART rates of virologic suppression were very high and similar in both groups, however, markers of inflammation (CRP and ferritin) remained significantly higher in those with co-infections.

In this study, we found a high prevalence of syphilis (14%) among ART-naïve PLWH. This result is comparable with previous studies that have estimated the prevalence of HIV–syphilis co-infection to be between 8% and 25%.^25,26^ We found that 10% of the participants were co-infected with HBV, 6% with HSV, and 3% with CMV. HIV–HBV co-infection has also been shown to be common, with studies showing a global prevalence of approximately 10% of PLWH due to shared routes of transmission.^27,28,29^ In Zimbabwe, HIV–HBV co-infection was found to be high (10%)^4^ and in a study in South Africa, it was reported to be 8.5% at baseline.^4,5^ The risk of liver cirrhosis, chronic hepatitis, and hepatocellular carcinoma is increased in HIV–HBV co-infection than in HBV mono-infection.^29,30,31^ In a systematic review consisting of eight SSA studies, the prevalence of CMV was higher (> 90%) in PLWH compared to uninfected counterparts.^6^ In the HPTN 071 (PopART) trial comprised of 4000 participants from Zambia and South Africa, HSV-2 and HIV were strongly associated.^7^

We found that participants with co-infections had significantly higher CRP and ferritin, but lower iron levels than those without co-infections at baseline. These findings confirm that the presence of co-infections in PLWH is associated with systemic inflammation. This is consistent with previous studies that showed that HIV co-infection with syphilis is associated with systemic inflammation characterised by elevations in CRP levels.^32^ The lower CD4 cell counts in participants with co-infections could be the consequence of immune activation.^33^ We found lower plasma iron levels at baseline in participants with co-infections, likely due to the pro-inflammatory state, which results in the liver releasing hepcidin which in turn signals the ferroportin channels to block the efflux of iron into circulation, resulting in reduced plasma iron levels.^34^

The current World Health Organization guidelines recommend a dolutegravir-based ART as the preferred first-line ART in low- and middle-income countries (LMICs) due to its high potency, decreased side effects, lower drug-to-drug interactions, and high genetic barrier to resistance.^35^ The present study supports the use of dolutegravir as the preferred first-line ART in many LMICs. The high potency of dolutegravir as previously reported^36,37,38^ was associated with 99% virological suppression (VL < 1000 copies/mL) after 6 months on ART in our study. These findings are consistent with previous findings from large clinical trials on virological efficacy of ART-naïve individuals receiving dolutegravir-based ART.^39,40,41^

Our study has some strengths, including the measurement of a broad range of biomarkers that include VL, CRP, iron, and ferritin, which were representative of viral replication, inflammation, and iron metabolism. In addition, the prospective nature of this study allowed us to demonstrate significant changes in various studied parameters following ART initiation. However, our study also had limitations. The main limitations of the current study include the small sample size and the fact that study participants were from a single primary healthcare centre, meaning that the interpretations may not be transferrable to the general population. Additionally, screening for other co-infections, such as tuberculosis or bacterial infections which increase CRP level, was not done among the participants. We also did not check for the presence or clearance of these co-infections at follow-up. We were also not certain if any participant developed other clinically relevant infections or if participants were given iron supplement during the follow-up period. A larger study over a longer study period with more data points on the clinical markers and description of the treatment would be more ideal to directly link the use of dolutegravir to reduced levels of inflammation in PLWH.

## Conclusion

This study showed that the presence of co-infections among PLWH was associated with increased inflammation, VL, and iron deficiency. However, following ART initiation, significant decreases in VL, CRP and ferritin, and increased iron levels, were observed. Early access to potent ART for effective management of HIV that includes lowering of VL, inflammation and stabilising plasma iron levels is therefore recommended.
